# Congenital absence of the left pericardium: a case report

**DOI:** 10.1186/s12872-023-03262-3

**Published:** 2023-05-12

**Authors:** Xiang-Yi Li, Yan Jiang, Hao-Wen Li, Yong-Kang Liu, Jing Bai

**Affiliations:** 1 Ultrasonic Department, China Aerospace Science & Industry Corporation 731 Hospital, No. 3, Zhen Gang Nan Li, Feng Tai District, 100074 Beijing, China; 2 AnoRectal Surgery, China Aerospace Science & Industry Corporation 731 Hospital, Beijing, China

**Keywords:** Pericardium, Absence of pericardium, Congenital heart defects, Case report

## Abstract

**Background:**

Congenital absence of the pericardium (CAP) is rare in clinical practice, the symptoms vary among patients, and most doctors do not have enough knowledge of the condition. Most reported CAP cases are incidental findings. Therefore, this case report aimed to present a rare case of left partial CAP that presented with non-specific, possibly cardiac-related symptoms.

**Case presentation:**

The patient, male, 56 years old, Asian, was admitted on March 2, 2021. The patient complained of occasional dizziness in the past week. The patient was suffering from hyperlipidemia and hypertension (stage 2), both untreated. The patient reported chest pain, palpitations, discomfort in the precordium, and dyspnea in the lateral recumbent position after strenuous activities, all of which started when he was about 15 years old. ECG showed sinus rhythm, 76 bpm, premature ventricular beats, incomplete right bundle branch block, and clockwise rotation of the electrical axis. Most of the ascending aorta could be detected in the parasternal intercostal space 2–4 by transthoracic echocardiography in the left lateral position. Chest computed tomography revealed the absence of pericardium between the aorta and the pulmonary artery, and part of the left lung was extending into the space. No changes in his condition have been reported up to now (March 2023).

**Conclusions:**

CAP should be considered when multiple examinations suggest heart rotation and a large moving range of the heart in the thoracic cavity.

**Supplementary Information:**

The online version contains supplementary material available at 10.1186/s12872-023-03262-3.

## Background

A congenital absence of the pericardium (CAP) is the rarest of all congenital heart defects, with a prevalence of 0.007-0.015% in autopsy series and 0.044% in a surgical case series [1–3]. CAP can present as the complete or partial absence of pericardium [1]. The worst complication of CAP is sudden cardiac death due to the strangulation of the heart across a partial defect of the left pericardium [1].

Most patients with CAP are asymptomatic and are incidentally discovered during lung or heart surgery, but some patients display unspecific symptoms that prompt investigations [1]. The symptoms are primarily due to the heart’s limited systolic and diastolic function caused by the incarceration of the heart and its appendages. CAP is rare in clinical practice, the symptoms vary among the patients, and most physicians do not have enough knowledge about the condition to diagnose and manage it properly. Hence, symptomatic patients can be misdiagnosed as having ischemic heart disease, leading to improper management [1, 4].

Most reported CAP cases are incidental findings in asymptomatic patients, as reviewed by Lopez et al. [1]. Therefore, this paper presents a rare case of left partial CAP with non-specific, possibly cardiac-related symptoms.

## Case presentation

The patient, male, 56 years old, Asian, office staff, went to the hospital for a physical examination on March 2, 2021. The patient complained of occasional dizziness in the past week. The patient was suffering from hyperlipidemia and hypertension (stage 2), both untreated. The patient reported chest pain, palpitations, discomfort in the precordium, and dyspnea in the lateral recumbent position after strenuous activities, which started when he was about 15 years old. His immediate family members shared no similar symptoms. Another hospital diagnosed a large moving range of the heart (by computed tomography (CT)) and congenital heart rotation (by echocardiography). The patient had been followed for 8 years at that hospital, but no intervention or treatment was given. The patient’s informed consent was obtained for this report.

**Fig. 1:**
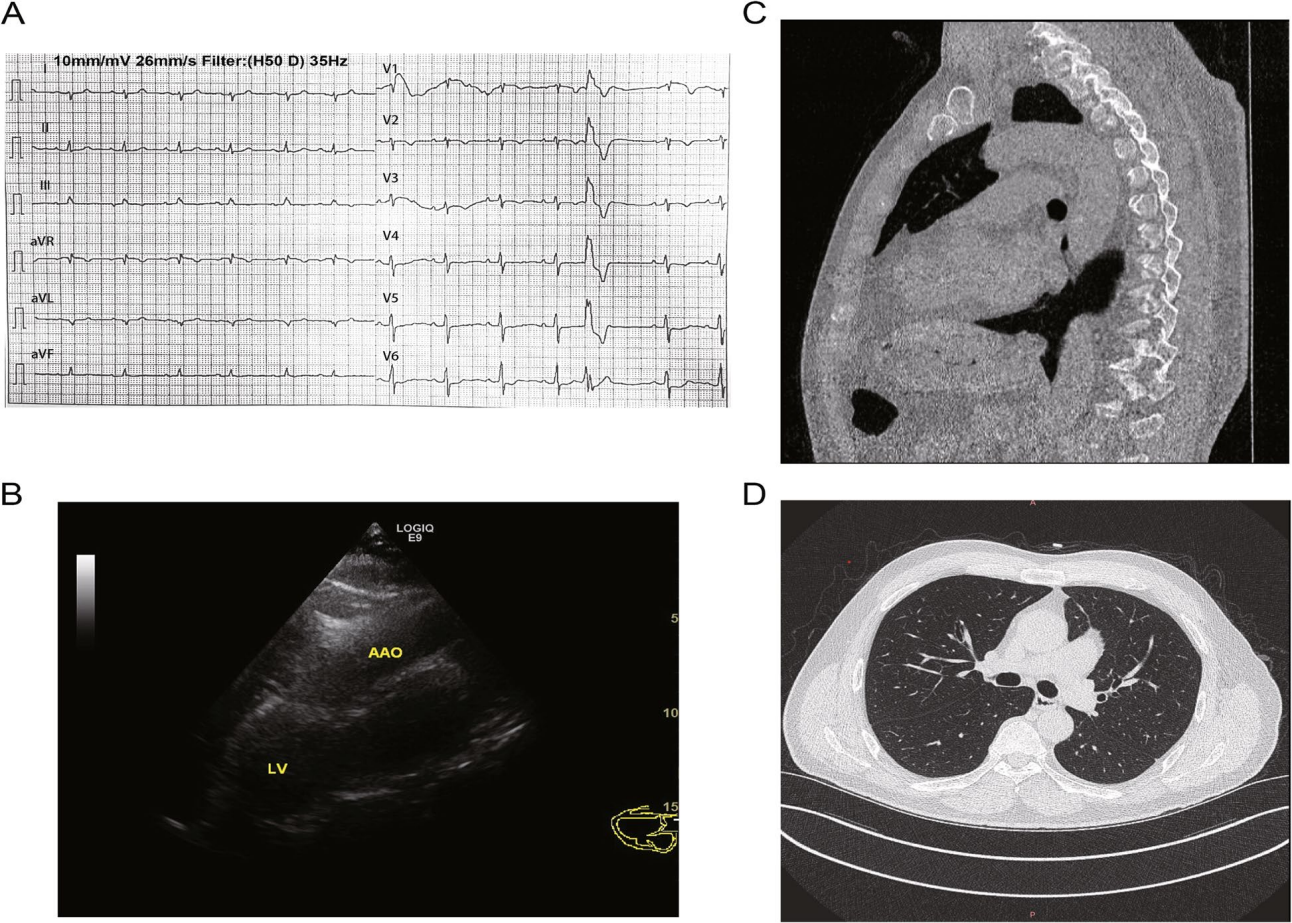
Results of the patient’s physical examination. **A** Electrocardiogram showing a sinus rhythm, 76 bpm, premature ventricular contractions, incomplete right bundle branch block, and clockwise heart rotation. **B** Conventional long-axis view of the parasternal left ventricle, showing most of the ascending aorta and part of the left ventricle. **C-D** There is no left pericardium image between the aorta and the pulmonary artery, and part of the left lung lobe extends into it

At physical examination, the strongest apical pulse was palpated in the left midaxillary line at the fifth intercostal space. ECG showed sinus rhythm, 76 bpm, premature ventricular beats, incomplete right bundle branch block, and clockwise rotation of the electrical axis (Fig. 1A). In normal echocardiography images, a long-axis section of the left ventricle should be observed between the left second, third, and four ribs in the parasternal position, including part of the right ventricle, the left ventricle excluding the apex, the left ventricular outflow tract, part of the ascending aorta, and the left atrium. Such normal images could not be obtained when the patient was in the left decubitus position (Fig. 1B). The left ventricular long-axis view was observed in the midaxillary position in the fourth intercostal space. Clockwise rotation of the electrical axis was detected when the patient was in the left lateral position. On the other hand, the heart was in a normal position when the examination was performed in the right lateral position, indicating a large range of movement in the chest. The heart structure was normal at rest, and the heart function parameters were in the normal range. Therefore, it was hypothesized that the absence of left pericardium might cause these signs. Chest CT revealed the absence of pericardium between the aorta and the pulmonary artery, and part of the left lung lobe extended into the space. Therefore, the patient was finally diagnosed with a congenital absence of the left pericardium (Fig. 1C-D).

The patient declined cardiac enhanced magnetic resonance (CMR) and angiography for financial reasons. Since the absence of the left pericardium had not significantly affected the patient’s work and life over the past 40 years, the patient decided to come to the hospital regularly for observation. The patient was given antihypertensive and lipid-lowering drugs and instructed to contact the hospital immediately if he felt uncomfortable.

The patient was followed up on May 10 and July 28, 2021. Up to now (March 2023), the patient reported no obvious increase or aggravation of symptoms. There are no changes in imaging or laboratory examination.

## Discussion and conclusions

CAP is the rarest congenital heart defect [1]. CAP is asymptomatic in most patients and is usually found incidentally during thoracic surgery or at autopsy [1–3]. Imaging is often unremarkable, and making a diagnosis is difficult. The patient reported here complained of unspecific symptoms, mostly dizziness. Combined with a previous diagnosis of a large moving range of heart and congenital heart rotation, more in-depth examinations were performed and finally suggested CAP, confirmed by CT looking for CAP. Although it is not the first case of CAP accompanied by unspecific symptoms, this case highlights that physicians should consider the possibility of CAP when multiple examinations suggest heart rotation and a large moving range of the heart in the thoracic cavity.

One of the most important functions of the pericardium is to hold the heart in place inside the chest. The concept of pericardium absence was first described in 1915 by Maude Abbott, a Canadian physician [1]. CAP is asymptomatic in most patients [1]. In patients undergoing cardiac surgery, the incidence of pericardium absence is about 0.044%, with more men than women, and of which, most were the absence of the left pericardium (63%), followed by the complete absence of the pericardium (37%) and the absence of the right pericardium (rarer) [2, 5]. Khayata et al. [6] conducted a retrospective study of eight patients with CAP and concluded that (1) relatively large defects of the left pericardium can lead to coronary artery insufficiency and heart and appendage herniation, and (2) medium-sized pericardium defects tend to lead to incarceration of the heart, resulting in limited cardiac activity, myocardial ischemia, syncope, and even death in the most serious cases. Therefore, for patients with cardiac symptoms, physicians must distinguish CAP from ischemic heart diseases to avoid misdiagnosis and provide the proper treatments.

The reported symptoms are usually atypical chest pain due to tension, pleuropericardial adhesions, lack of the pericardium cushioning effect, and torsion and tension of the great vessels due to heart movement and lack of heart support [1, 4]. In the presence of a large pericardium defect, the left lung and heart can coexist in the same pleural cavity, as observed in the case reported here, and symptoms may appear when the left lung compresses the heart and the aorta. It is also possible that the torsion and tension of the great vessels cause hemodynamic disturbances that could result in dizziness. The ECG changes (premature ventricular beats, incomplete right bundle branch block, and clockwise rotation of the electrical axis) could also explain the dizziness. In the case reported here, since dizziness was not interfering with work and daily life, it was not further investigated.

The imaging diagnosis of CAP is difficult. In the present case, CT showed that the heart was shifted to the left, and the X-ray transmittance between the ascending aorta and the main pulmonary artery was increased. In some cases, due to the rotation of the heart, the shape is an “enlarged heart shadow”, and it is difficult to observe the pericardium on echocardiography. In the case reported here, a normal cardiac image could not be obtained using the conventional standard view in the left lateral position, but the cardiac ultrasound showed most of the aorta and a small part of the left ventricle, while in the supine or right lateral position, normal images could be obtained. It is inferred from these indirect signs that the heart could move when the patient was in different positions, suggesting the lack of support and a possible absence of pericardium. Therefore, these indirect signs are needed for comprehensive analysis. In the case reported here, chest CT showed that the left lung was protruding between the ascending aorta and the main pulmonary artery because of the lack of pericardium, called the Snoopy Sign [7]. CMR examination is the gold standard for the Snoopy Sign, but it can also be observed using CT [7]. Although CMR is the gold standard for diagnosing CAP, CMR is not available in all hospitals. Without CMR, an echocardiogram and chest CT can provide clues for diagnosing CAP.

Due to the rarity of the disease, no management guidelines exist. For small to medium-sized pericardial absence, preventive surgery can be used to repair the pericardium or enlarge the defect to avoid heart herniation and incarceration [1, 4, 5, 8]. On the other hand, some authors believe that the risk of postoperative infection overcomes the potential benefits of surgery [1, 4]. The patient reported here was 56, and the symptoms were not interfering with his work and daily life. Observation was selected.

In conclusion, the patient reported a 40-year history of chest pain, palpitations, discomfort in the precordium, and dyspnea in the lateral recumbent position after strenuous activities, an 8-year history of heart rotation and large range motion, and a 1-week history of dizziness. Echocardiography suggested moving heart in the chest. CT revealed the absence of pericardium. The take-home message of this report is that physicians should consider the possibility of CAP when the patient reports non-specific, possibly cardiac-related symptoms and when different examinations suggest heart rotation and movement in the thoracic cavity.

## Supplementary Information

**Additional file 1.**

**Additional file 2.**

**Additional file 3.**

**Additional file 4. **

**Additional file 5.**

## Data Availability

The datasets used and/or analyzed during the current study are available from the corresponding author upon reasonable request.

## Abbreviations

CAP: Congenital absence of the pericardium
ECG: Electrocardiogram
CT: Computed tomography
MRI: Magnetic resonance imaging
CMR: Cardiac enhanced magnetic resonance
