# Supplementary material for: Congenital absence of the left pericardium: a case report
Source: BMC Cardiovasc Disord. 2023 May 12;23:247. doi: 10.1186/s12872-023-03262-3 (PMC10176923; doi:10.1186/s12872-023-03262-3)
Supplement: Supplementary file 2 — Additional file 2. [file 12872_2023_3262_MOESM2_ESM.pdf]

informed consent

Dear patient:

The disease that you have right now is The left pericardium was absent , Is a rare congenital malformation. We invite you to participate in a clinical study and participation is entirely your choice. Please be careful to make your decision to participate in the study. If you have any questions about the study, you can ask your doctor or researcher to explain them. You can discuss with your family and friends, and you have the right to refuse to participate in this study, or to withdraw from the study at any time without punishment or losing your rights.

This study is a case follow-up, and if you decide to participate in this study, you must come to the hospital at the agreed follow-up time between your doctor and you. Your follow-up is very important. During the follow-up period, the investigators will use relevant clinical observation forms to collect all your observation data. You will receive meticulous assessment, monitoring and treatment beyond routine monitoring, potential improvement in your

condition, and this study may help with other patients with similar conditions.

Your participation in the study and your personal data during the study are kept confidential. Information that can identify you will not be disclosed to members outside of the study team without your permission. Your profile is available to researchers only. To ensure that the study is conducted in accordance with the regulations, members of the government administration or the ethics review committee can access your personal data at the study site if necessary. No personal information of you will be disclosed on publication of the results of this study.

Patient (subject) Name:

宋斌

date: 2021.4.10
