# Supplementary material for: Congenital absence of the left pericardium: a case report
Source: BMC Cardiovasc Disord. 2023 May 12;23:247. doi: 10.1186/s12872-023-03262-3 (PMC10176923; doi:10.1186/s12872-023-03262-3)
Supplement: Supplementary file 3 — Additional file 3. [file 12872_2023_3262_MOESM3_ESM.docx]

**Title:** Congenital absence of the left pericardium: A case report

**Manuscript ID:** BCAR-D-22-00483

**Journal:** BMC Cardiovascular Disorders

**Response to Reviewers' comments**

Dear Editor,

We thank you for your careful consideration of our manuscript. We appreciate your response and overall positive initial feedback and made modifications to improve the manuscript. After carefully reviewing the comments made by the Reviewers, we have modified the manuscript to improve the presentation of our results and their discussion, therefore providing a complete context for the research that may be of interest to your readers.

We hope that you will find the revised paper suitable for publication, and we look forward to contributing to your journal. Please do not hesitate to contact us with other questions or concerns regarding the manuscript.

Best regards,

Yan Jiang

**Editor**

***Comment 1:*** CARE guidelinesIn accordance with BioMed Central editorial policies(http://www.biomedcentral.com/submissions/editorialpolicies#standards+of+reporting), could you please ensure your manuscript reporting adheres to CARE guidelines (http://www.care-statement.org/downloads) for reporting of case reports. This is so your methodology can be fully evaluated and utilized. Can you please include a completed CARE checklist as an additional file when submitting your revised manuscript. Please complete the checklist in full by inserting the page number/paragraph and section of your manuscript which reports the information that meets the criteria of the checklist. For example, "Methods, paragraph 2". If a criterion is not applicable for your particular manuscript/study, we can accept "N/A". Please note that checklists completed incorrectly will be returned for revision as we cannot progress your manuscript to peer review until the checklist has been completed.

**Response:** We thank for the comment. The checklist was already completed.

***Comment 2:*** Consent for publication- Please note that we require written informed consent to be obtained for all case reports. As indicated in our editorial policies, "For all manuscripts that include details, images, or videos relating to an individual person, written informed consent for the publication of these details must be obtained from that person (or their parent or legal guardian in the case of children under 18)." Consent to publish may also be obtained from the next of kin under appropriate circumstances. Please confirm whether consent to publish was obtained for this study. If consent for publication was not obtained, we would like to ask if you are able to go back and obtain this consent retrospectively. Please be advised that the consent form must be made available to the Editor if requested, and will be treated confidentially.

**Response:** We thank for the comment. We provided the informed consent as a separate file.

***Comment 3:*** At this stage, please upload your manuscript as a clean version that does not contain any tracked changes, comments, highlights, strikethroughs or text in different colors.

**Response:** We thank for the comment. The manuscript is clean now.
