# Supplementary material for: Congenital absence of the left pericardium: a case report
Source: BMC Cardiovasc Disord. 2023 May 12;23:247. doi: 10.1186/s12872-023-03262-3 (PMC10176923; doi:10.1186/s12872-023-03262-3)
Supplement: Supplementary file 4 — Additional file 4. [file 12872_2023_3262_MOESM4_ESM.docx]

**Title:** Congenital absence of the left pericardium: A case report

**Manuscript ID:** BCAR-D-22-00077R2

**Journal:** BMC Cardiovascular Disorders

**Response to Reviewers' comments**

Dear Editor,

We thank you for your careful consideration of our manuscript. We appreciate your response and overall positive initial feedback and made modifications to improve the manuscript. After carefully reviewing the comments made by the Reviewers, we have modified the manuscript to improve the presentation of our results and their discussion, therefore providing a complete context for the research that may be of interest to your readers.

We hope that you will find the revised paper suitable for publication, and we look forward to contributing to your journal. Please do not hesitate to contact us with other questions or concerns regarding the manuscript.

Best regards,

**Reviewer #1**

***Comment 1:*** *I am very sorry, but this case report does not add anything to the literature to be deserved to be published.*

**Response:** We thank the Reviewer for the comment. Congenital absence of the pericardium (CAP) is the rarest congenital heart defect [1]. It is asymptomatic in the vast majority of patients and is usually found incidentally during thoracic surgery or at autopsy [1]. As presented in the Discussion, even the imaging diagnosis is difficult. The patient reported here complained of some unspecific symptoms (dizziness). Combined with a personal history of a diagnosis of a large moving range of heart and congenital heart rotation, more in-depth examinations were performed and suggested CAP, which was confirmed by imaging examinations looking for CAP. This case highlights that physicians should consider the possibility of CAP when multiple examinations suggest heart rotation and a large moving range of the heart in the thoracic cavity. It was clarified in the Discussion.

***Comment 2:*** *The relationship of dizziness to* *partial congenital absence of pericardium is not discussed.*

**Response:** We thank the Reviewer for the comment. CAP is asymptomatic in the vast majority of patients [1], and the reported symptoms are usually atypical chest pain that can be due to tension from pleuropericardial adhesions, lack of the pericardium cushioning effect, and torsion and tension of the great vessels due to a lack of support and heart movement [1-3]. It is also possible that the torsion and tension of the great vessels cause hemodynamic disturbances that could result in dizziness. The ECG changes (premature ventricular beats, incomplete right bundle branch block, and clockwise rotation of the electrical axis) could also lead to some degree of dizziness. Partial CAP is also associated with trepopnea, i.e., dyspnea in specific positions due to the position of the heart and lungs in specific positions, and subclinical dyspnea for long periods can lead to dizziness. Still, since the dizziness was not interfering with work and daily life, it was not further investigated. It was added to the Discussion.

***Comment 3:*** *The Figures are of poor quality.*

**Response:** We thank the Reviewer for the comment. We now provide figures with a higher resolution. Please note that the figures we are now providing are at the highest resolution allowed by our equipment.

***Comment 4:*** *A chest radiograph of the patient with findings of partial congenital absence of pericardium is not included. Usually, chest X-ray shows very helpful findings in this regard*

**Response:** We thank the Reviewer for the comment. We agree that a chest X-ray is a simple examination that is often overlooked despite the fact that it can provide a wealth of useful information. The patient did not have a chest X-ray, and we think CT is much clearer than a chest X-ray and can be further reconstructed in three-dimensions CT if necessary.

***Comment 5:*** *The authors could include more recent papers in the references section instead of references belonging to 1993, 1996, and 2000.*

**Response:** We thank the Reviewer for the comment. We updated the references. Still, considering the rarity of the condition, older references can be necessary.

**Reviewer #2**

***Comment 1:*** *This is a good case report. Congenital absence of the pericardium (CAP) is rare in clinical practice.This case reports can be used to highlight the physicians who should consider the possibility of CAP when multiple examinations suggest heart rotation and a large moving range of the heart in the thoracic cavity.There is a minor suggestion for your echocardiography image.Please provide more higher resolution images.*

**Response:** We thank the Reviewer for the comment. We now provide figures with a higher resolution. Please note that the figures we are now providing are at the highest resolution allowed by our equipment.

**References**

1.	Lopez D, Asher CR. Congenital Absence of the Pericardium. Prog Cardiovasc Dis. 2017;59(4):398-406. Epub 2016/12/14. doi: 10.1016/j.pcad.2016.12.002. PubMed PMID: 27956198.

2.	Newman B. Congenital Absence of the Pericardium: Pearls and Pitfalls. Semin Ultrasound CT MR. 2022;43(1):47-50. Epub 2022/02/16. doi: 10.1053/j.sult.2021.05.005. PubMed PMID: 35164909.

3.	Nicholas J, Barber E, Grondin S, Haber J. Congenital absence of the pericardium encountered during thoracoscopy. Can J Anaesth. 2022;69(7):898-9. Epub 2022/04/20. doi: 10.1007/s12630-022-02250-z. PubMed PMID: 35437684.
