# Supplementary material for: Congenital absence of the left pericardium: a case report
Source: BMC Cardiovasc Disord. 2023 May 12;23:247. doi: 10.1186/s12872-023-03262-3 (PMC10176923; doi:10.1186/s12872-023-03262-3)
Supplement: Supplementary file 5 — Additional file 5. [file 12872_2023_3262_MOESM5_ESM.docx]

**Title:** Congenital absence of the left pericardium: A case report

**Manuscript ID:** BCAR-D-22-00483R3

**Journal:** BMC Cardiovascular Disorders

**Response to Reviewers’ comments**

Dear Editor,

We thank you for your careful consideration of our manuscript. We appreciate your response and overall positive feedback and made modifications to improve the manuscript.

We hope that you will find the revised paper suitable for publication, and we look forward to contributing to your journal. Please do not hesitate to contact us with other questions or concerns regarding the manuscript.

Best regards,

**Reviewer 1**

***Comment 1:*** *What is the novelty of this case report? This is a rare entity, as you mentioned many times in the article and your response, but there are many published case reports and I don’t see any* *novelty in the presentation of your patient. Please clarify this.*

**Response:** We thank the Reviewer for the comment. Yes, the goal of case reports is to report completely novel and previously unreported medical conditions, but case reports are also used to present very rare medical conditions that are poorly understood and very sketchily described in the literature and textbooks to help physicians around the globe when they encounter such rare cases.

The patient reported here was complaining of unspecific symptoms, mostly dizziness. Combined with a previous diagnosis of a large moving range of heart and congenital heart rotation, more in-depth examinations were performed and finally suggested CAP, which was confirmed by CT looking for CAP. Although it is not the first case of CAP accompanied by unspecific symptoms, this case highlights that physicians should consider the possibility of CAP when multiple examinations suggest heart rotation and a large moving range of the heart in the thoracic cavity.

***Comment 2:*** *The case presentation is unclear. Why patient came after 15 years of same symptoms?*

**Response:** We thank the Reviewer. The patient had been symptomatic since he was 15 years old. CAP was diagnosed at 56 years. The patient had symptoms for 41 years, but the symptoms were not interfering with his daily life.

***Comment 3:*** *The name and localization of hospital where patient was admitted and diagnosed should be omitted as it is not important for the article.*

**Response:** It was deleted.

***Comment 4:*** *The authors stated that “another hospital diagnosed a large moving range of heart and congenital heart rotation”. How was this diagnosed?*

**Response:** We thank the Reviewer for the comment. The large moving range was diagnosed by CT, and the heart rotation was diagnosed by echocardiography. It was clarified in the manuscript.

***Comment 5:*** *What does it mean “echoes of most of the ascending aorta could be detected in the parasternal intercostal space 2-4”? The description of echocardiographic examination sound very strange and it is not clear. Please ask cardiologist for clarification, rephrase the whole paragraph and add clips in order to explain your findings.*

**Response:** We thank the Reviewer for the comment. In normal echocardiography images, a long-axis section of the left ventricle should be observed between the left second, third, and four ribs in the parasternal position, including part of the right ventricle, the left ventricle excluding the apex, the left ventricular outflow tract, part of the ascending aorta, and the left atrium. Such normal images could not be obtained when the patient was in the left decubitus position. It was clarified in the manuscript.

***Comment 6:*** *Please add ECG tracings in different body positions in order to illustrate differences.*

**Response:** We thank the Reviewer for the comment. Unfortunately, that examination was not performed.

***Comment 7:*** *The quality of images is poor and your explanation that machines are not providing better* *resolution is not acceptable. Your images look like print screen instead of images taken from corresponding programs or PACS. This is not sufficient for publication.*

**Response:** We thank the Reviewer for the comment. We now provide higher-quality images.

***Comment 8:*** *The authors mentioned that CMR and angiography were recommended, but I did not understand the explanation why these investigations (particularly CMR) were not performed.*

**Response:** The Reviewer is entirely right. The patient was indeed recommended to undergo CMR, but he declined for financial reasons. It was clarified in the manuscript.

***Comment 9:*** *Why cardiac enzymes were recommended for follow-up? What is happening with patient now? We are in January 2023…*

**Response:** We thank the Reviewer for the comment. The purpose of checking myocardial enzyme levels is to monitor for eventual ischemic events or infarction due to local myocardial entrapment. Up to now (March 2023), the patient reported no obvious increase or aggravation of symptoms. There are no changes in imaging or laboratory examination. It was clarified in the manuscript.

***Comment 10:*** *Discussion is too long and not focused. Please change this.*

**Response:** We thank the Reviewer for the comment. We revised the Discussion.

***Comment 11:*** *Conclusion is too short and not informative. Please correct this.*

**Response:** We thank the Reviewer for the comment. We expanded the Conclusion: “In conclusion, the patient reported a 40-year history of chest pain, palpitations, discomfort in the precordium, and dyspnea in the lateral recumbent position after strenuous activities, an 8-year history of heart rotation and large range motion, and a 1-week history of dizziness. Echocardiography suggested a moving heart in the chest. CT revealed the absence of the pericardium. The take-home message of this report is that physicians should consider the possibility of CAP when the patient report non-specific, possibly cardiac-related symptoms and when different examinations suggest heart rotation and movement in the thoracic cavity.”

***Comment 12:*** *Editing of English is necessary. Please ask native English speaker to help you.*

**Response:** We thank the Reviewer. The manuscript was proofread.
